# Estimation of MERS-Coronavirus Reproductive Number and Case Fatality Rate for the Spring 2014 Saudi Arabia Outbreak: Insights from Publicly Available Data

**DOI:** 10.1371/currents.outbreaks.98d2f8f3382d84f390736cd5f5fe133c

**Published:** 2014-12-18

**Authors:** Maimuna S. Majumder, Caitlin Rivers, Eric Lofgren, David Fisman

**Affiliations:** Engineering Systems Division, Massachusetts Institute of Technology, Cambridge, Massachusetts, USA; Network Dynamics and Simulation Science Laboratory, Virginia Bioinformatics Institute, Virginia Tech, Blacksburg, VA, USA; Network Dynamics and Simulation Science Laboratory, Virginia Bioinformatics Institute, Virginia Tech, Blacksburg, Virginia, USA; Dalla Lana School of Public Health, University of Toronto, Toronto, Ontario, Canada

## Abstract

Background: The Middle East Respiratory Syndrome Coronavirus (MERS-CoV) was initially recognized as a source of severe respiratory illness and renal failure in 2012. Prior to 2014, MERS-CoV was mostly associated with sporadic cases of human illness, of presumed zoonotic origin, though chains of person-to-person transmission in the healthcare setting were reported. In spring 2014, large healthcare-associated outbreaks of MERS-CoV infection occurred in Jeddah and Riyadh, Kingdom of Saudi Arabia. To date the epidemiological information published by public health investigators in affected jurisdictions has been relatively limited. However, it is important that the global public health community have access to information on the basic epidemiological features of the outbreak to date, including the basic reproduction number (R0) and best estimates of case-fatality rates (CFR). We sought to address these gaps using a publicly available line listing of MERS-CoV cases. Methods: R0 was estimated using the incidence decay with exponential adjustment (“IDEA”) method, while period-specific case fatality rates that incorporated non-attributed death data were estimated using Monte Carlo simulation. Results: 707 cases were available for evaluation. 52% of cases were identified as primary, with the rest being secondary. IDEA model fits suggested a higher R0 in Jeddah (3.5-6.7) than in Riyadh (2.0-2.8); control parameters suggested more rapid reduction in transmission in the former city than the latter. The model accurately projected final size and end date of the Riyadh outbreak based on information available prior to the outbreak peak; for Jeddah, these projections were possible once the outbreak peaked. Overall case-fatality was 40%; depending on the timing of 171 deaths unlinked to case data, outbreak CFR could be higher, lower, or equivalent to pre-outbreak CFR. Conclusions: Notwithstanding imperfect data, inferences about MERS-CoV epidemiology important for public health preparedness are possible using publicly available data sources. The R0 estimated in Riyadh appears similar to that seen for SARS-CoV, but CFR appears higher, and indirect evidence suggests control activities ended these outbreaks. These data suggest this disease should be regarded with equal or greater concern than the related SARS-CoV.

## Introduction

On September 20, 2012, the discovery of a novel human coronavirus was announced on ProMED 1. The virus, later named Middle East Respiratory Syndrome Coronavirus (MERS-CoV), was isolated from a 60-year-old patient in the Kingdom of Saudi Arabia, who presented in June 2012 with pneumonia and acute renal failure 1 ^,^ 2 . On September 23, ProMED announced that two more cases had been diagnosed in London, one of whom had traveled to Saudi Arabia in August 3 ^,^ 4 . Two of those three initial cases died, raising questions about the potential public health implications of this new virus 3.

Although apparent instances of sustained secondary transmission did occur in healthcare-related outbreaks prior to early 2014, MERS-CoV was until this time regarded as a primarily zoonotic pathogen with limited potential for person-to-person spread. In spring 2014, a large outbreak originated in the Kingdom of Saudi Arabia – concentrated in healthcare facilities in Riyadh and Jeddah – resulting in over 300 infections and approximately 40 deaths, as well as considerable domestic and international concern. Nearly two years and an outbreak later, many of the questions raised about MERS in the early days of its discovery still remain. Of significant concern is the pandemic potential of the disease, as well as its true case fatality rate.

To date, several modeling studies have estimated the basic reproduction number to be near or below the critical pandemic threshold of R_0_> 1 4 ^,^ 5 ^,^ 6 ^,^ 7 . However, most of these estimates fail to account for the large numbers of asymptomatic or mild cases, nor for the public health response to recognized clusters, both of which resulted in reduced transmission of MERS-CoV during the Spring 2014 outbreak. In this sense, the models published thus far describe effective, rather than basic, reproductive numbers. This distinction is important, as it is the basic reproduction number that determines epidemic potential 5. Furthermore, the character of outbreaks in healthcare settings suggests that while the overall reproduction number for this pathogen may be low, there is heterogeneity in transmission based on setting, and reproduction numbers in the healthcare context are likely to be higher than those seen in the community and household settings 5 .

The virulence of the pathogen, as distinct from its transmissibility, also remains uncertain. While overall case fatality rate can be estimated for MERS-CoV, it is unclear whether CFR was elevated in the context of the spring 2014 outbreak. Public reporting on MERS cases by the Kingdom of Saudi Arabia and other affected countries has been limited. Case history gaps, lags in reporting, and sudden “dumps” of novel data have contributed to considerable uncertainty about key epidemiological properties of this disease. Because of the status of most-impacted countries as global hubs for business, tourism, and religious pilgrimage, and the prior occurrence of disease importation into countries lacking putative MERS-CoV reservoirs, better understanding of MERS-CoV epidemiology and epidemic dynamics is a global need. To address outstanding questions in the absence of detailed official data sources, we used publicly available data to study the dynamics and epidemiology of MERS-CoV infection. Our objectives were to:

1. Describe the basic epidemiological features of the outbreak to date;
2. Approximate the basic reproduction number (R_0_) of the disease in the context of the Spring 2014 outbreak, in a manner that accounted for extensive disease control activities by affected localities;
3. Estimate non-outbreak and outbreak case fatality rates after adequately addressing missing outcome data using Monte Carlo simulation.

## Methods

Data Source

A line listing of MERS-CoV cases maintained by Andrew Rambaut was accessed updated on June 2, 2014 and accessed on June 4, 2014 8 . The line list was compiled from publicly available sources, including the Kingdom of Saudi Arabia (KSA) Ministry of Health and World Health Organization reports. A "frozen" version of the dataset used for the analyses described below is available at http://figshare.com/articles/MERS_data_June_4_2014/1243671. The list contained 707 cases worldwide, which agreed well with the 699 laboratory-confirmed cases announced by the World Health Organization on June 11, 2014 9 . Five hundred and ninety of those cases occurred in Saudi Arabia; however, because the list only provides outcome information at time of original reporting, the Rambaut data only account for 116 deaths in Saudi Arabia (case fatality rate [CFR] = 19.7%) while the Saudi Ministry of Health listed 195 deaths as of June 2 (CFR = 33.0%). Furthermore, the Rambaut list does not contain an additional 113 cases and 92 deaths announced on June 3 that were retrospectively identified by the Saudi Ministry of Health 11 .

***Estimation of Basic Reproductive Number (R***_0_ **)**

From March to June 2014 nosocomial outbreaks of MERS infections occurred in the Saudi cities of Jeddah and Riyadh. We, and others, have previously estimated basic reproductive numbers (R_0_) for MERS based on cluster size estimates. However, because those methods provide solutions when R_0_ < 1, cluster-based estimates have predictably been < 1 4 ^,^ 5 ^,^ 6 . The outbreaks in Jeddah and Riyadh had typical “bell-shaped” epidemic curves , suggesting exponential growth with initial R > 1. We used publicly available data to generate time-varying estimates of R_0_ using a previously described single equation method: the incidence decay and exponential adjustment model (“IDEA”) model 12 .

The IDEA model is based on the observation that in the absence of control measures, the number of incident cases in an epidemic generation (t) will be equivalent to R_0_ *^t^*. However, because control efforts are implemented after the outbreak is recognized, the effective R will also decay exponentially, a phenomenon that appears to be empirically well-represented by the quantity I_t_ = [R_^o^_/(1+d)^t^]^t^. For low or moderate *R_0_* this approach provides estimates concordant with those produced by compartmental mathematical models with exponential reductions in transmission due to control.We estimated parameters (*R_0_* and *d*) by fitting the model to incidence and cumulative incidence epidemic curves using the Berkeley Madonna software package (Berkeley, California, http://www.berkeleymadonna.com/).

The IDEA model is parameterized in terms of epidemic generations rather than calendar time. Consequently, case counts were aggregated based on serial intervals. For the purposes of model fitting, the Spring 2014 outbreak was assumed to start (Generation 1) with the identification of sequential serial generations with non-zero case counts. In other words, we examined time periods downstream from cases, aggregated by serial intervals as described below, and if > 2 sequential serial intervals contained aggregated case counts > 0 an outbreak was considered to have begun. Consequently, outbreak start date was considered to be March 2014 (Riyadh, March 5, 2014; Jeddah, March 16, 2014).

In contrast, other analyses below assigned the Spring 2014 outbreak to periods with incidence elevated significantly above baseline (i.e., April to June 2014). Only 44% of cases in the data had reported onset date. Significant differences from the mean were seen in lags from onset to reporting in secondary cases and in those with comorbidity (P < 0.05 for all comparisons). Thus, we assumed that onset dates were missing at random, but not completely at random 13 . Missing onset dates for symptomatic cases were replaced probabilistically using tabular data from March 2014 onwards, with dates replaced separately based on comorbidity and secondary case status. In other words, we created tables of the probability of a given lag from onset to report for each of four possible combinations of comorbidity and secondary case classes, and replaced missing lags for individuals with each combination using random draws from these tabular probability distributions.

Following the estimates of Assiri et al 14 , we performed model fits for both Riyadh and Jeddah using 6-, 7- and 8-day serial intervals. Initial fits were performed using data as of June 2, 2014. Additional analyses to evaluate how early in the outbreak models could be fit to the data were performed using only the first three generations of each city. Subsequent models were fit using a progressively larger number of generations.

Imputation of Case-Fatality Rates Including 171 Non-Attributable Deaths

Because the Saudi Ministry of Health often announces deaths after initial case reporting – without any formal referral mechanism to the original case listing – it remains unclear whether MERS-related CFR during the Spring 2014 outbreak (considered April 2014 – Early June 2014, based on elevated case counts) differed significantly from months previous. As of June 2, 2014, 590 Saudi cases and 116 Saudi deaths were accounted for in the line list. Then, on June 3, the Saudi Ministry of Health announced the addition of 113 previously unreported cases – and 92 previously unreported deaths – to the cumulative tally 11 . An epidemic curve of case onset dates by month was also published 11 , but the curve did not depict death dates. After digitizing the epidemic curve, these 113 cases were added to the 590 in the line list; sorted by dates of onset, hospitalization, or reporting; and grouped by month, resulting in a 703 cases total.

The Saudi Ministry of Health reported a total of 282 deaths on June 3, but only 116 were listed in the dataset 11 . 5 of these 116 listed deaths occurred among exported cases and thus were not documented by the Saudi Ministry of Health 8 ; as a result, 171 deaths emerged as non-attributable. We distributed these 171 deaths over four varying time frames (Simulations 1 – 4) using Monte Carlo simulations of N = 1000 to determine how different imputations would impact pre-outbreak and through-outbreak CFR. In all four simulations:

1. Distribution of non-attributable deaths was proportional to the number of monthly cases specified in the “distribution timeframe” column of Table 1;
2. The random number generator for monthly deaths was parametrized using 0 (deaths) as the minimum and the number of listed non-fatal cases as the maximum; and
3. The simulated deaths from March 2012 to June 2014 were set to sum at 117 for each of the 1000 runs (simulated outbreaks).

For each month, the mean number of simulated deaths was calculated using randomly generated data from all 1000 runs; these mean values were then added to the listed deaths to create a “total fatal” variable. This variable was then used to calculate mean estimates of pre-outbreak and through-outbreak CFR for each simulation.

**Table 1. Description of simulation parameters (number of deaths distributed and timeframe over which deaths were distributed) and overview of simulation results (mean pre-outbreak CFR and mean through-outbreak CFR).** Each simulation was run N = 1000 times. Pre-outbreak and through-outbreak periods are defined as March 2012 – March 2014 and April 2014 – June 2014, respectively.

Simulation	Randomly Distributed Deaths	Distribution Timeframe	Mean Pre-Outbreak Case Fatality Rate	Mean Through-Outbreak Case Fatality Rate
1	171	March 2012—June 2014	51%	36%
2	171	April 2014—June 2014	30%	46%
3	171	June 2013—June 2014	50%	36%
4	171	January 2014—June 2014	38%	42%

## Results

Descriptive Epidemiology

The Arabian Peninsula was the epicenter of the Spring 2014 MERS outbreak. Of the 707 cases listed in the Rambaut data set as of June 2, 2014, 84% were infected in Saudi Arabia, and 98% were infected in the Arabian Peninsula. Cases acquired in other regions (e.g. UK, France) via secondary transmission all had an epidemiological connection to a person recently returned from the Arabian Peninsula. Men were overrepresented among cases (61.5%, N=686) and deaths (72.6%, N=139). The median age of all cases was 47 (N=698) and 60 among deaths. 40% of cases had a reported comorbidity (e.g., diabetes), and 17.1% of cases were reported as healthcare workers. One third (35.5%) of cases in women were reported as mild or asymptomatic, compared with 24.1% of cases in men. It is unknown whether women were more likely to experience less severe disease, or whether they had a different exposure source associated with milder illness. Over half (51.6%) of cases in the Rambaut data were listed as primary cases, possibly acquired from an animal source or an unknown infected contact. Several family and healthcare-associated clusters brought the number of secondary cases to 342 (48.4%).

Estimation of Basic Reproductive Number (R_0_)

Models fit using all available generations from March 1 to June 2 showed excellent agreement with outbreak data for 6, 7, and 8 day generation times (Figure 1).

**Concordance between model fits (curves) and cumulative incidence data for Jeddah (circles) and Riyadh (squares).**
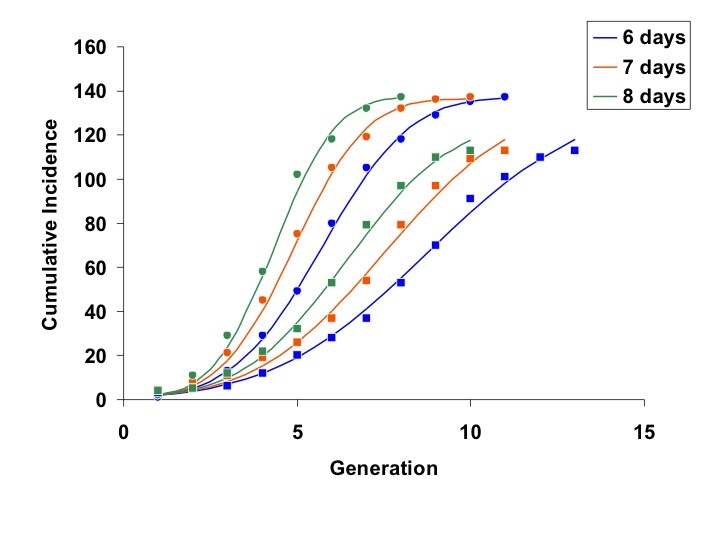
Generations can be converted to calendar dates as follows: the final day of each generation may be estimated as ((outbreak start date – 1) + generation x serial interval). Start date is March 5, 2014 for Riyadh and March 16, 2014 for Jeddah, such that (for example) the final day of generation 2 for Riyadh, using a 6-day serial interval, is (March 4, 2014 + 2 x 6 = March 16, 2014).

Best-fit R_0_ estimates for the Riyadh outbreak ranged from 2.0 to 2.8, regardless of serial interval used. Estimates for the Jeddah outbreak were higher, ranging from 3.5 to 6.7. Similarly, the corresponding estimates for *d* were lower and more stable for Riyadh than for Jeddah (0.04 to 0.09, and 0.13 to 0.28, respectively) (Figure 2).

**Best fit estimates of reproductive number ((R), left-side panel), and the model “discount factor” ((d), right-sided panel), for Riyadh (blue bars) and Jeddah (red bars).**
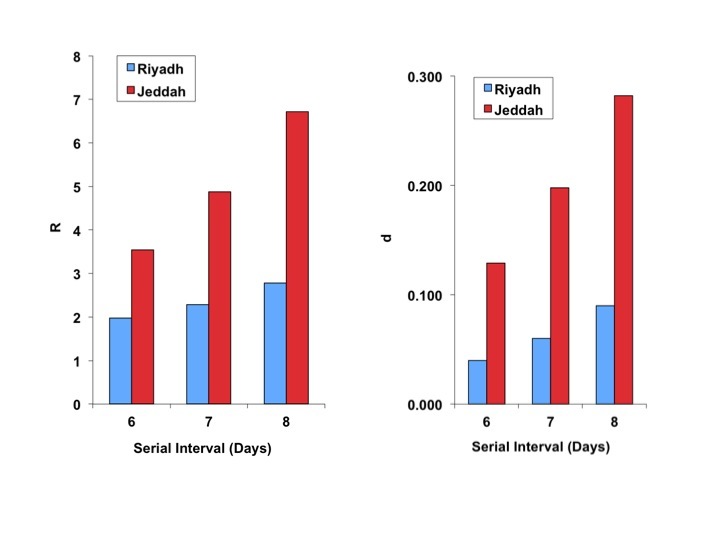
As expected, R estimates increase with increasing serial interval for both cities, though the increase is more marked for Jeddah. Both *R* and *d* are higher for Jeddah, denoting a more explosive, but more aggressively controlled, outbreak.

Taken together, these data suggest a more explosive outbreak in Jeddah, with correspondingly more intensive and effective control efforts. As the IDEA method is scaled in generation time, the effective reproductive number (Re) in some generation *t* can be approximated as *I_t_/I_(t-1)_*. The estimated Re for the Riyadh and Jeddah outbreaks, over time, are plotted in Figure 3.

**Effective Reproductive Numbers Over Time, Riyadh and Jeddah Outbreaks:**
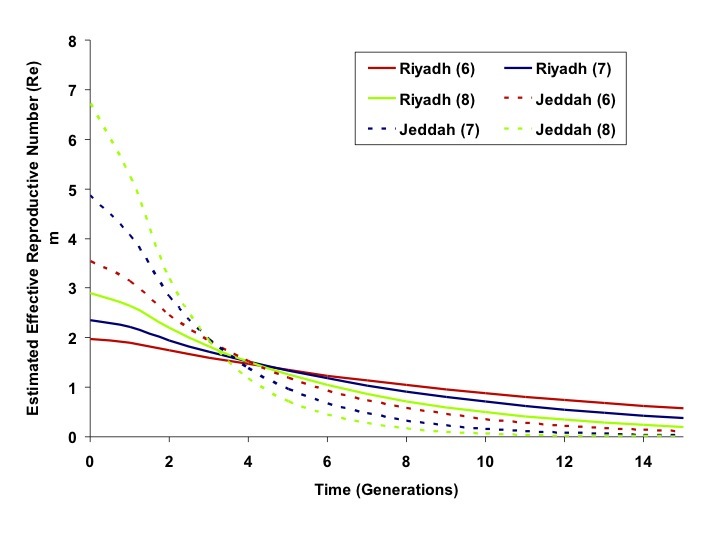
Estimated effective reproductive numbers (*R_e_*) for outbreaks in Riyadh (solid curves) and Jeddah (dashed curves) based on 6, 7 or 8 day generation times (red, blue, and green, respectively). *R_e_* is initially higher in Jeddah but decreases more rapidly than in Riyadh.

When we used progressively increasing numbers of generations for model fits, we found that the full course of the outbreak (including the magnitude and timing of the outbreak peak, outbreak end date, and total outbreak size) could be predicted with reasonable accuracy before the outbreak had peaked for the city of Riyadh. Projects generated using a 6-day serial interval predicted the full extent of the Riyadh outbreak reasonably well by the fourth generation of the outbreak; best-fit estimates of *R_0_* and *d* derived using these data changed negligibly as further generations were included (Figure 4).

**Best-fit estimates for model parameters R (top figures) and d (bottom figures), using sequentially increasing numbers of outbreak generations for both Riyadh (left-side panels) and Jeddah (right-side panels).**
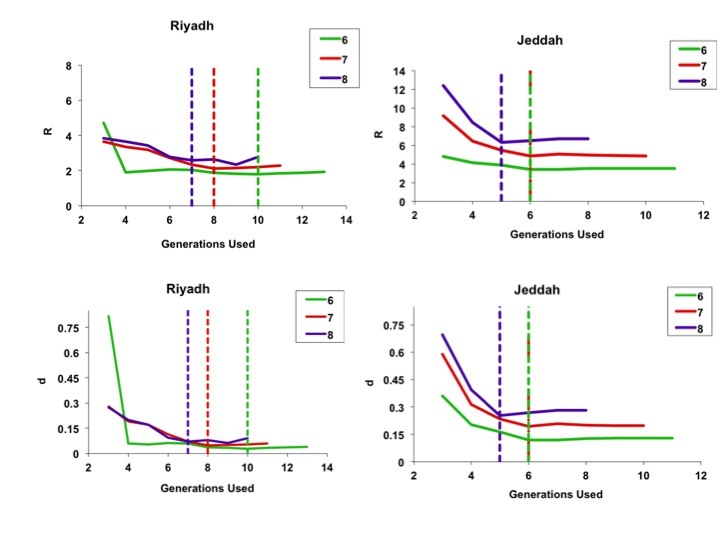
Color coding denotes 6-, 7-, or 8-day serial intervals as identified in figure legends. For the less explosive Riyadh outbreak, parameter estimates appear to stabilize rapidly when fits used only a few initial outbreak generations, whereas the more explosive Jeddah outbreak parameters appeared to stabilize once data up to and including the outbreak peak were included. Dashed lines denote the timing (by generation) of the outbreak peaks.

As demonstrated in Figures 5 and 6, curves generated by fitting to the full outbreak time series differed little from those that could be generated by fitting to only the initial 6 or 8 generations of data (the outbreak peaked in generation 9).

**Modelled Incidence Curves based on Models Fit to Varying Numbers of Generations, Riyadh (6 Day Generation Time):**
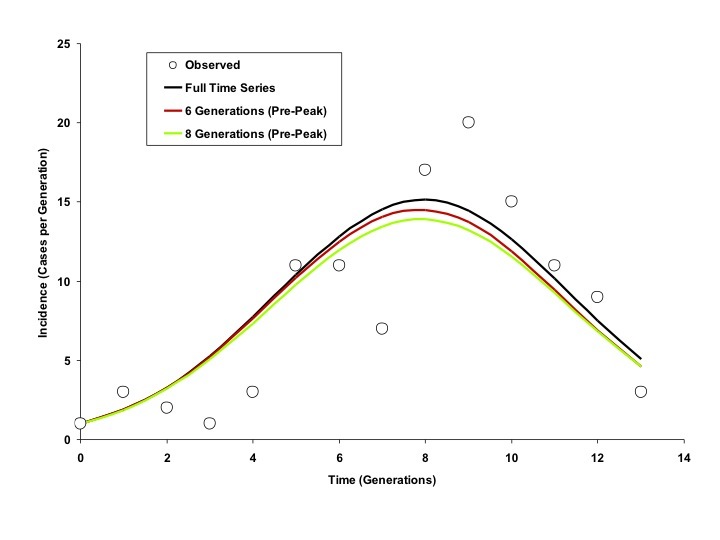
Circles represent observed per-generation incidence. The dark blue curve is generated using an IDEA model fit to the entire time series. The red and green curves represent IDEA models fit using only 6 or 8 outbreak generations; in other words, these are outbreak projections based on case counts prior to the outbreak peak. Curves generated using pre-peak data are minimally different from those generated using the full time series.

**Modelled Cumulative Incidence Curves based on Models Fit to Varying Numbers of Generations, Riyadh (6 Day Generation Time):**
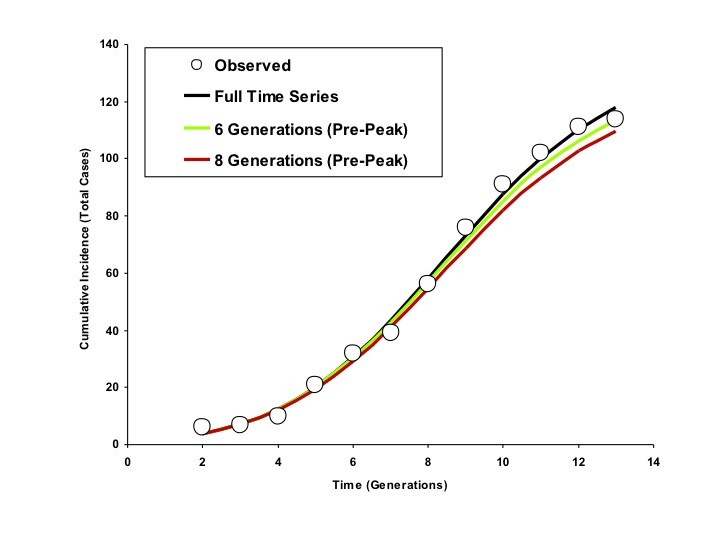
Circles represent observed cumulative incidence by generation. The dark blue curve is generated using an IDEA model fit to the entire time series. The red and green curves represent IDEA models fit using only 6 or 8 outbreak generations (cumulative incidence prior to the outbreak peak). Curves generated using pre-peak data are minimally different from those generated using the full time series.

By contrast, for the more explosive Jeddah outbreak, parameter estimates stabilized only when the epidemic peaked. This finding is consistent with our earlier published simulations showing that IDEA fits accurately predict the full extent of high *R_0_* outbreaks only when time series up to and including peak incidence and subsequent decline are used for fitting 12(Figures 7 and 8).

**Modelled Incidence Curves based on Models Fit to Varying Numbers of Generations, Jeddah (6 Day Generation Time):**
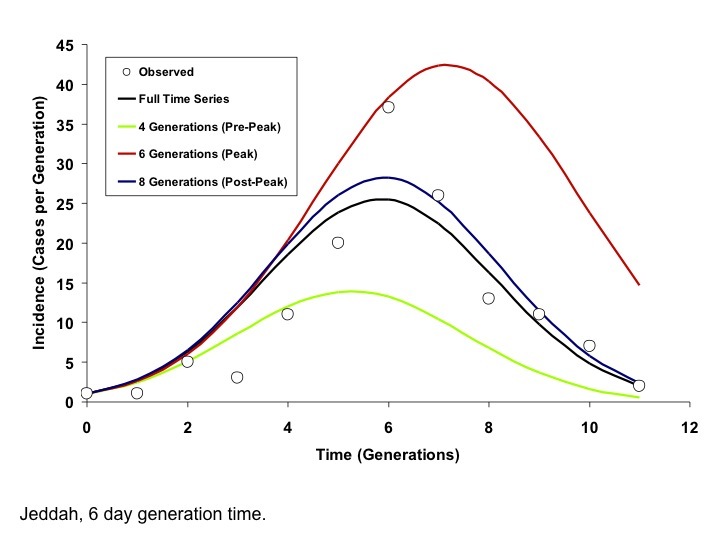
Circles represent observed per-generation incidence. The dark blue curve is generated using an IDEA model fit to the entire time series. The red and green curves represent IDEA models fit using only 4 or 6 outbreak generations; prior to or at the outbreak peak, respectively. The black curve is generated by fitting the model to 9 generations of data, encompassing the outbreak peak and initial decline. The post-peak curve and full time series curve are almost identical, but the full contour of the outbreak is difficult to project based on earlier data.

**Modelled Cumulative Incidence Curves based on Models Fit to Varying Numbers of Generations, Jeddah (6 Day Generation Time):**
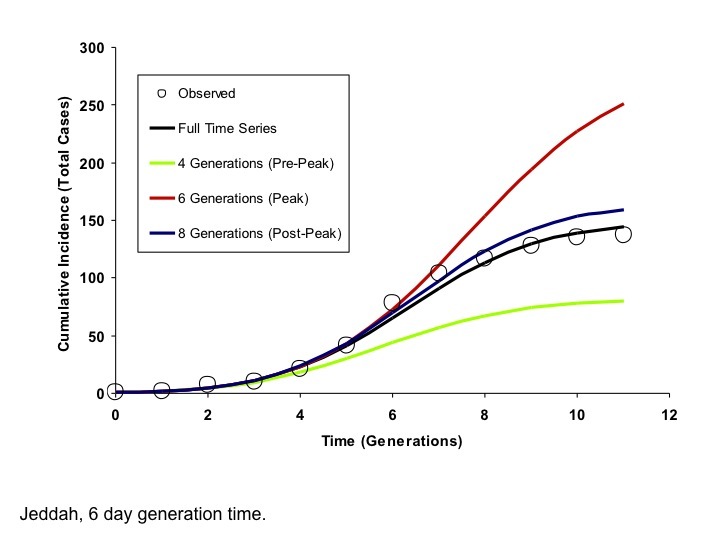
Circles represent observed cumulative incidence by generation. The dark blue curve is generated using an IDEA model fit to the entire time series. The red and green curves represent IDEA models fit using only 4 or 6 outbreak generations, prior to or at the outbreak peak, respectively. The black curve is generated by fitting the model to 9 generations of data, encompassing the outbreak peak and initial decline. The post-peak curve and full time series curve are almost identical, but the full contour of the outbreak is difficult to project based on earlier data.

Imputation of Case-Fatality Rates Including 117 Non-Attributable Deaths

As shown in Table 1, each of the four simulations distributed the 171 non-attributable Saudi MERS deaths over different time periods. The mean results of the four simulations are visualized in Figure 9.

**Stacked epicurves of non-fatal and fatal cases for Simulations 1 – 4.**
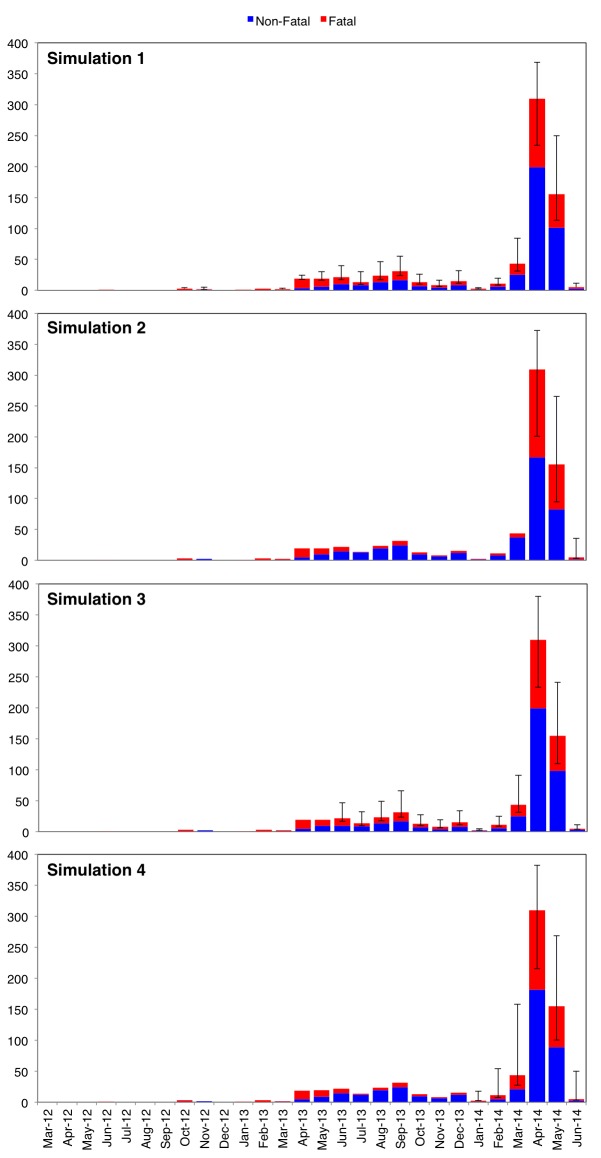
Each simulation was run N = 1000 times. Colored bars represent mean values for fatal (red) and non-fatal (blue) cases by month over 1000 runs. Error bars indicate minimum and maximum values for fatal cases by month over 1000 runs. In all 4 simulations, 171 non-attributable deaths were randomly distributed across different periods of time: all months (March 2012 – June 2014) for Simulation 1; only outbreak months (April 2014 – June 2014); only months from the year preceding the outbreak (June 2013 – June 2014); and only months in 2014 (January 2014 – June 2014).

**Table 2. Comparison of mean pre-outbreak and mean through-outbreak simulation CFR.** Each simulation was run N = 1000 times. Two-tailed chi-square with Yates’ correction was used for statistical testing. Pre-outbreak and through-outbreak periods are as defined above.

Simulation	Period	Mean Fatal Cases	Mean Non-Fatal Cases	Mean Case Fatality Rate	χ^2^-statistic	P-value
1	Pre-Outbreak	119	115	51%	14	0.0002
	Through-Outbreak	168	302	36%		
2	Pre-Outbreak	69	165	30%	18	<0.001
	Through-Outbreak	218	252	46%		
3	Pre-Outbreak	117	117	50%	12	0.0006
	Through-Outbreak	170	299	36%		
4	Pre-Outbreak	89	145	38%	0.92	0.3372
	Through-Outbreak	198	272	42%		

The degree of difference between mean CFR under pre-outbreak and through-outbreak conditions is largely contingent on the distribution timeframe selected (Table 2). In Simulations 1 and 3, mean through-outbreak CFR is significantly less than mean pre-outbreak CFR (p = 0.0002 and p = 0.0006, respectively). However, in Simulation 2, mean through-outbreak CFR is significantly greater than mean pre-outbreak CFR (p < 0.0001). Meanwhile, in Simulation 4, the mean pre/through-outbreak CFR measures are not significantly different from one another – suggesting only negligible fluctuation in fatality (p = 0.3372).

Until each of the 171 non-attributable deaths can be tied to their respective cases, it will be difficult to determine conclusively whether the Spring 2014 outbreak CFR was indeed less than (Simulations 1 and 3), equal to (Simulation 4), or greater than (Simulation 2) what we have seen in the past. However, if we assume that non-attributable deaths were equally likely to occur among pre-outbreak cases and through-outbreak cases, Simulation 1 serves as an appropriate mean estimate. In the event that this assumption is true, the CFR among Saudi cases during the Spring 2014 outbreak (36%) may have been substantially less than in months prior (51%).

## Discussion

Understanding the epidemiological attributes of a novel, emerging infectious disease allows clinicians and public health practitioners to implement efficient, effective disease control interventions that reduce the size and impact of an outbreak or epidemic. However, rapid study of an infectious disease is always difficult, and even more so in an environment where geographic, jurisdictional, informational or political factors make population-based research challenging. While the occurrence of MERS-CoV infection in humans has now apparently been occurring for over two years, high-quality epidemiological analyses from countries with the greatest burden of MERS-CoV cases are still in short supply. Although local acquisition of MERS-CoV has occurred overwhelmingly on the Arabian Peninsula, this disease is a global public health concern and it is important for clinicians and public health practitioners both inside and outside the most-impacted regions to have sufficient information for formulation of good preventive practices.

In the absence of detailed official data sources from health authorities in affected countries, we used publically available data to estimate two key epidemiological parameters that are critical to the formulation of good public health policy: the basic reproduction number (R_0_) of the disease and its case-fatality rate (CFR), under outbreak conditions. These parameters define both the gravity of a disease, as well as its potential for ongoing transmission. Perhaps unsurprisingly, given our investigation in the context of an outbreak, we found estimates of R_0_ that are higher than those previously reported in the literature, which themselves were based on sporadic case occurrence and self-limited clusters 4 ^,^ 6 ^,^ 7 . Furthermore, most of the estimates we derived were in the 2-3 range, similar to that estimated for SARS-CoV in the context of hospital outbreaks 16 ^,^ 17 . Estimates for Jeddah were even higher, though this elevated R_0_ was accompanied by a high d, suggesting an explosive outbreak which was brought under control relatively rapidly. Taken together, these observations suggest that the healthcare environment represents fertile soil for the transmission of MERS-CoV, as it did for SARS-CoV, and that measures instituted in response to SARS (including strengthening hospital infection control programs) would also be effective against MERS.

Our second major insight relates to the virulence [MM1] of MERS-CoV under outbreak and non-outbreak conditions. While one might expect case-fatality to be higher in the context of an outbreak concentrated in healthcare facilities, our simulated imputations of non-attributable deaths suggest that this may not have been the case during the Spring 2014 MERS outbreak. Assuming that the probable occurrence of non-attributable death was relatively uniform between March 2012 and June 2014, CFR may have in fact been significantly less (p = 0.0002) during the Spring 2014 outbreak (36%) than during non-outbreak months (51%). This suggests that cases during the outbreak may have been characteristically different with respect to mortality risk factors from cases that occurred prior to the outbreak.

While the CFR associated with the outbreak was calculated only among recognized cases, (a “denominator” for this calculation would require seroprevalence studies to assess the true cumulative incidence of MERS-CoV infection) this is an extraordinarily high CFR by any standard. By way of context, it should be noted that the 2002-2003 SARS outbreak had an estimated CFR of 17%. These findings emphasize the need for continued vigilance and the development of treatment protocols for those infected with MERS-CoV.

As with any observational epidemiological study, ours has limitations, which include data quality, assumptions necessary for model building, and the potential impact that under-recognition of mild and asymptomatic MERS-CoV cases may have had on our epidemiological estimates. To some extent, concern regarding the impact of such limitations on our results can be assuaged by the fact that findings remained robust in the face of alternate assumptions and evaluation of different scenarios. Of note, we do not regard the fact that we have used public, rather than official, data sources for the performance of these analyses as a major limitation. A recent paper by Lau and colleagues evaluated the concordance between publicly available datasets, and official governmental datasets in the context of the recent influenza A (H7N9) outbreak in China 18. In addition to a high degree of concordance between public and official data sources, the authors founds that publicly available data sources have the further advantage of permitting epidemiological analyses in a timely and transparent manner.
